# Human DDX3 Interacts with the HIV-1 Tat Protein to Facilitate Viral mRNA Translation

**DOI:** 10.1371/journal.pone.0068665

**Published:** 2013-07-01

**Authors:** Ming-Chih Lai, Shainn-Wei Wang, Lie Cheng, Woan-Yuh Tarn, Shaw-Jenq Tsai, H. Sunny Sun

**Affiliations:** 1 Department of Physiology, National Cheng Kung University Medical College, Tainan, Taiwan; 2 Institute of Molecular Medicine, National Cheng Kung University Medical College, Tainan, Taiwan; 3 Institute of Basic Medical Sciences, National Cheng Kung University Medical College, Tainan, Taiwan; 4 Institute of Biomedical Sciences, Academia Sinica, Taipei, Taiwan; Institut National de la Santé et de la Recherche Médicale, France

## Abstract

Nuclear export and translation of intron-containing viral mRNAs are required for HIV-1 gene expression and replication. In this report, we provide evidence to show that DDX3 regulates the translation of HIV-1 mRNAs. We found that knockdown of DDX3 expression effectively inhibited HIV-1 production. Translation of HIV-1 early regulatory proteins, Tat and rev, was impaired in DDX3-depleted cells. All HIV-1 transcripts share a highly structured 5’ untranslated region (UTR) with inhibitory elements on translation of viral mRNAs, yet DDX3 promoted translation of reporter mRNAs containing the HIV-1 5’ UTR, especially with the transactivation response (TAR) hairpin. Interestingly, DDX3 directly interacts with HIV-1 Tat, a well-characterized transcriptional activator bound to the TAR hairpin. HIV-1 Tat is partially targeted to cytoplasmic stress granules upon DDX3 overexpression or cell stress conditions, suggesting a potential role of Tat/DDX3 complex in translation. We further demonstrated that HIV-1 Tat remains associated with translating mRNAs and facilitates translation of mRNAs containing the HIV-1 5’ UTR. Taken together, these findings indicate that DDX3 is recruited to the TAR hairpin by interaction with viral Tat to facilitate HIV-1 mRNA translation.

## Introduction

Human DDX3 belongs to the DEAD-box protein family of RNA helicases, which are characterized by nine conserved motifs that mediate ATP hydrolysis and RNA helicase activities. It has been reported that DDX3 functions in many aspects of mRNA metabolism, including transcription [1,2], pre-mRNA splicing, mRNA transport [3,4] and translation initiation [5–9]. The role of DDX3 in translation initiation seems to be evolutionarily conserved from yeast to humans [10]. In the fission yeast *Schizosaccharomyces pombe*, DDX3 homolog Ded1 is implicated in the translational control of two B-type cyclins, Cig2 and Cdc13 *[11]* ***.*** Notably, *Cig2* mRNA has an unusually long 5’ untranslated region (UTR), while the 5’ UTR of *Cdc13* mRNA is predicted to contain a complex secondary structure. Consistent with this notion, we recently demonstrated that DDX3 facilitates the translation of mRNAs with a long or structured 5’ UTR [7,8]. The RNA helicase activity of DDX3 is required for its function in translation. Therefore, DDX3 and its homologs may facilitate translation initiation by resolving secondary structures in the 5’ UTR of selected mRNAs.

Human immunodeficiency virus type 1 (HIV-1) is a complex retrovirus that causes acquired immune deficiency syndrome (AIDS). The genome of HIV-1 is diploid and consists of two identical positive-stranded RNA molecules that can be reverse transcribed into double-stranded DNA and integrated into the host genome. HIV-1 gene expression and replication are regulated at the transcriptional and post-transcriptional steps. HIV-1 transcription yields a single ~9kb pre-mRNA that undergoes alternative splicing to generate over 40 different mRNA species. In the early phase of HIV-1 replication, only fully spliced viral mRNAs that encode three regulatory proteins (Tat, Rev and Nef) are exported to the cytoplasm via the TAP-mediated export pathway [12]. The HIV-1 Tat protein is a well-characterized transcriptional activator that specifically binds to the transactivation response (TAR) hairpin within the 5’ UTR of all HIV-1 transcripts and recruits cyclin T-CDK9 to RNA polymerase II [13–15]. The HIV-1 Rev protein is a key switch from the early to late phase of HIV-1 replication. HIV-1 Rev binds to the cis-acting Rev response element (RRE) present in unspliced viral genomic RNA and partially spliced mRNAs, which primarily encode viral structural proteins (Gag and Env). Typically, the nuclear export of intron-containing mRNAs is severely restricted in mammalian cells. To ensure successful replication, HIV-1 has evolved the Rev/RRE/CRM1-mediated pathway for the export of intron-containing viral mRNAs to the cytoplasm [12]. Previously, DDX3 has been reported to function as an accessory factor in the Rev/RRE/CRM1-mediated export of HIV-1 mRNAs [16,17]. DDX3 was shown to facilitate nuclear export of RRE-containing viral mRNAs, but not cellular mRNAs, via interactions with HIV-1 Rev and cellular export receptor CRM1 [16,17]. Depletion of cellular DDX3 by RNA interference exerted suppressive effects on HIV-1 replication [18], suggesting that DDX3 could be a potential therapeutic target for anti-HIV therapy [19,20].

Translation of eukaryotic mRNAs is generally initiated by ribosome scanning [21]. This mode of initiation involves binding of the 40S ribosomal subunit together with translation initiation factors to the 5’ cap of mRNAs and subsequent scanning for the AUG start codon. Secondary structures near the 5’ cap have been shown to inhibit translation initiation by interfering with ribosome scanning [22]. HIV-1 and related retroviruses contain a highly conserved 5’ UTR, which is essential for gene expression and viral replication [23]. All HIV-1 transcripts, whether they are spliced or not, share the same highly structured 5’ UTR *[24]* ***.S***everal structural elements within the HIV-1 5’ UTR are thought to be involved in translational control [25]. However, the molecular mechanisms underlying the control of HIV-1 mRNA translation remain poorly understood.

A large number of reports have provided convincing evidence that DDX3 and its homologs play important roles in translation [5–11,26–31]. More recently, it has been demonstrated that DDX3 and its homologs function as a key protein in the formation of cytoplasmic stress granules (SGs) [9,26], supporting a bona fide role of DDX3 in translational control. Although the requirement of DDX3 for the export of intron-containing HIV-1 mRNAs has been documented [16,18], the involvement of DDX3 in HIV-1 mRNA translation remains to be deciphered. In this study, we provide evidence to demonstrate that DDX3 interacts with the HIV-1 Tat protein and controls viral mRNA translation.

## Materials and Methods

### Plasmid construction

The pSilencer 1.0-U6 (Ambion) vector was used to express shRNAs targeting human DDX3 (sh-DDX3#1: coding region nucleotides 433-451; sh-DDX3#2: coding region nucleotides 1699-1717; sh-DDX3#3: coding region nucleotides 131-149). The construct of HIV-1 proviral DNA plasmid (pHXB2gpt) has been described [32]. The pEGFP-N1 (Clontech) encoding GFP protein serves as a control for transfection efficiency. For *in vivo* translation assay, the reporter pFL-SV40 has been described previously [7]. The full-length and partial HIV-1 5’ UTR DNA fragments were obtained by PCR using the pHXB2gpt plasmid as template. Each 5’ UTR DNA fragment was inserted into an unique HindIII site upstream of the firefly luciferase coding region in the pFL-SV40 vector. All constructs were confirmed by direct sequencing. The plasmid expressing HIV-1 Tat (pTat) has been described [33]. To generate histidine (His)-tagged recombinant proteins, the open reading frames of HIV-1 Tat and Rev were amplified by PCR using pTat and pcRev [34] plasmids as templates and inserted in-frame into the pET-15b (Novagen) vector with appropriate restriction enzymes. The plasmids expressing GST-DDX3 and a series of truncated DDX3 fragments have been described in detail [1]. The plasmids expressing GFP-DDX3, shRNA-resistant wild-type DDX3 and S382L mutant have been described previously [7].

### Cell culture and transfection

HeLa and human embryonic kidney 293 (HEK293) cells were grown at 37^°^C in Dulbecco’s modified Eagle medium (DMEM) supplemented with 10% fetal bovine serum, 100 U/ml penicillin and 100 µg/ml streptomycin. Cell transfection was performed using Lipofectamine 2000 (Invitrogen), essentially according to the manufacturer’s instructions.

To measure HIV-1 production efficiency, HeLa cells were seeded in 6-well culture dishes (2 × 10^5^ cells/well) and transfected with indicated shRNA-expressing vectors (2 µg). After 36 hours, cells were re-transfected with the proviral DNA plasmid pHXB2gpt (1.5 µg), pEGFP-N1 (1 µg) and the same shRNA-expressing vectors (1.5 µg). Transfected cells were cultured for another 48 hours and harvested in 2% SDS buffer. Total protein was resolved by 10% SDS-PAGE, followed by immunoblotting with the indicated antibodies.

### Sucrose gradient fractionation

Cells were collected in cold PBS containing 100 µg/ml cycloheximide. All subsequent steps were performed at 4^°^C. Cell pellets were resuspended in RSB-150 (10 mM Tris-HCl (pH 7.4), 3 mM MgCl_2_, and 150 mM NaCl) containing 100 µg/ml cycloheximide, 40 µg/ml digitonin (Calbiochem), 20 U/ml RNasin (Promega) and 1× protease inhibitor cocktail (Thermo Scientific). After incubation on ice for 5 min, cells were disrupted by passage through a 26-gauge needle five times. Cytoplasmic extracts were collected by centrifugation at 3,000 × *g* for 2 min, and clarified by further centrifugation at 11,000 × *g* for 15 min. The samples were loaded on a linear 15-40% sucrose gradient and centrifuged at 38,000 rpm for 3 h in a Beckman SW41 rotor. After centrifugation, total RNA was extracted from each fraction using phenol/chloroform extraction in the presence of 1% SDS and 0.25 M NaCl, followed by ethanol precipitation. For immunoblot analysis, total protein was precipitated with 10% trichloroacetic acid (TCA) and then washed with cold acetone. Precipitated proteins were solubilized in 1× SDS sample buffer.

### Reverse transcription-polymerase chain reaction (RT-PCR)

RT-PCR was used to detect the mRNA expression level. Extracted RNA was reverse-transcribed into cDNA using the High-Capacity cDNA Reverse Transcription Kits (Applied Biosystems) according to manufacturer’s instructions. The resulting cDNA was subjected to conventional PCR or quantitative real-time PCR analyses. Conventional PCR was performed using GoTaq DNA polymerase (Promega) according to the manufacturer’s instructions. The primers and their sequences used for PCR analyses were: HIV-1 Tat (Forward: 5’-ATGGAGCCAGTAGATCCTAG-3’ and Reverse: 5’-TATTCCTTCGGGCCTGTCG-3’), HIV-1 Rev (Forward: 5’-CGAC GAAGAGCTCATCAGAAC-3’ and Reverse: 5’-GGTAGCTGAAGAGGCACAGG- 3’), β-actin (Forward: 5’-CATCCACGAAACTACCTTCAACT-3’ and Reverse: 5’-TCTCCTT AGAGAGAAGTGGGGTG-3’).

Quantitative real-time PCR was carried out on the ABI Prism GeneAmp 7900 HT (Applied Biosystems) according to suppliers’ recommendations. The primers and TaqMan probes were designed using the Custom TaqMan, Assay Design Tool (Applied Biosystems). Quantitative analysis was performed by the measurement of CT values during the exponential phase of amplification. All experiments were repeated at least three times.

#### Subcellular fractionation

Cells were collected in cold PBS containing 100 µg/ml cycloheximide. To obtain the cytoplasmic fraction, cells were resuspended in cold buffer A (10 mM HEPES (pH 7.9), 10 mM KCl, 1.5 mM MgCl_2_, 0.5 mM DTT, and 5% glycerol) containing 20 U/ml RNasin (Promega) and 1× protease inhibitor cocktail (Thermo Scientific). After incubation on ice for 15 min, add Triton X-100 to a final concentration of 0.1% and place the sample on ice for 2 min. Extracts were centrifugated at 3,000× g for 5 min at 4^°^C. The supernatants were collected as the cytoplasmic fraction. The pellets were resuspended in cold buffer B (20 mM HEPES (pH 7.9), 450 mM NaCl, 1.5 mM MgCl_2_, 0.2 mM EDTA, 0.5 mM DTT, and 25% glycerol) containing 20 U/ml RNasin (Promega) and 1× protease inhibitor cocktail (Thermo Scientific). Place the sample on ice and continue vortexing for 15 seconds every 10 min. After incubation on ice for 30 min, extracts were centrifugated at maximum speed for 10 min at 4^°^C. The supernatants were collected as the nuclear fraction. Total RNA was purified from each fraction using TRIzol LS reagent (Invitrogen).

### Translation assays

For *in vivo* translation assay, HeLa cells (2 × 10^5^ cells/well) were co-transfected with a pFL-SV40 derived reporter (0.2 µg), the control pRL-SV40 vector (0.2 µg) and a shRNA-expressing vector (1 µg). For the DDX3 rescue assay, a vector expressing shRNA-resistant DDX3 (0.6 µg) was used for co-transfection. At 48 h post-transfection, cells were lysed in 1× Passive Lysis Buffer (Promega). The activities of firefly luciferase and *Renilla* luciferase were measured using the Dual-Luciferase Reporter Assay System (Promega).

The *in vitro* translation assay was performed using the 1-Step Human IVT Kit-mRNA (Thermo Scientific) according to the manufacturer’s instructions. The *in vitro*-transcribed HIV-1 5’ UTR-containing reporter mRNA (0.1 µg) and control reporter mRNA (0.1 µg) were used as templates. The translation reaction was incubated at 30^°^C for 2 h in the absence or presence of recombinant HIV-1 Tat.

#### *In* *vitro* pull-down assay

His-tagged recombinant HIV-1 Tat (0.4 µg) or HIV-1 Rev (0.4 µg) protein was incubated with 2 µg GST or GST-DDX3 fusion protein in a 60-µl mixture at 30^°^C for 30 min. The reaction mix was supplemented with an equal volume of NET-2 buffer (50 mM Tris-HCl (pH 7.4), 150 mM NaCl, and 0.05% Nonidet P-40) and subsequently incubated with 10 µl of Glutathione Sepharose 4B (GE Healthcare) at 4^°^C for 2 h. After extensive washing with NET-2 buffer, bound proteins were eluted with 1× SDS sample buffer and resolved by 12% SDS-PAGE, followed by immunoblotting with anti-HIV-1 Tat or anti-HIV-1 Rev antibody. GST-fusion proteins were detected by Coomassie blue staining.

### Immunoprecipitation and immunoblotting

HIV-1 Tat was expressed alone or co-expressed with FLAG-tagged DDX3 in HEK293 cells (5 × 10^6^) for 48 h. Immunoprecipitation of FLAG-tagged DDX3 was performed according to the method described by Lykke-Andersen et al. [35]. Cell lysates (0.5 ml) prepared from transfected HEK293 cells were incubated with 10 µl of anti-FLAG M2-agarose (Sigma) at 4^°^C for 2 h. To immunoprecipitate HIV-1 Tat, two microliters of anti-HIV-1 Tat antibody (Abcam) were coupled to protein A sepharose beads in NET-2 buffer (50 mM Tris-HCl (pH 7.4), 150 mM NaCl, and 0.05% Nonidet P-40). The beads were washed four times with 1 ml of NET-2 buffer to remove unbound proteins. Immunoprecipitates were treated with 1 mg/ml RNase A at 37^°^C for 30 min. Bound proteins were eluted with 1× SDS sample buffer and resolved by 12% SDS-PAGE.

For immunoblot analysis, proteins were transferred onto a PVDF Transfer Membrane (PerkinElmer). Protein blots were blocked with 3% skim milk in TBST buffer (100 mM Tris-HCl (pH 7.6), 150 mM NaCl, and 0.05% Tween 20) at RT for 1 h. The primary antibodies used included mouse anti-HIV-1 Tat (0.1 µg/ml; Santa Cruz Biotechnology), affinity-purified rabbit anti-DDX3 (0.1 µg/ml) [7], mouse anti-HIV-1 Rev (1 µg/ml; Abcam), rabbit anti-eIF4A1 (1 µg/ml; Abcam), mouse anti-eIF2α (0.4 µg/ml; Santa Cruz Biotechnology), rabbit anti-eIF4E (2 µg/ml; Abcam), mouse anti-HIV1 p24 (1 µg/ml; Abcam), rabbit anti-α-tubulin (1:2000 dilution; Cell Signaling), mouse anti-lamin A/C (0.2 µg/ml; Santa Cruz Biotechnology), and rabbit anti-GFP (0.2 µg/ml; Abcam). Blots were incubated with primary antibodies in blocking buffer at RT for 2 h, followed by incubation with HRP-conjugated secondary antibodies at RT for 2 h. Detection was performed using Immobilon Western Chemiluminescent HRP Substrate (Millipore) for X-ray film exposure.

### Immunofluorescent staining

HIV-1 Tat was expressed alone or co-expressed with GFP-DDX3 in HeLa cells for 24 h. Immunofluorescent staining of HeLa cells was performed as described previously [36]. Briefly, cells grown on cover slips were rinsed with cold PBS and fixed with 3% formaldehyde in PBS for 30 min, followed by permeabilization with 0.5% Triton X-100 in PBS for 10 min. After washing with PBS, cells were blocked with 1% BSA in PBS for 30 min and then incubated with the relevant primary antibodies in PBS at RT for 1 h. The primary antibodies used were mouse anti-HIV-1 Tat (0.4 µg/ml; Santa Cruz Biotechnology), goat anti-TIA-1 (2 µg/ml; Santa Cruz Biotechnology), and rabbit anti-eIF4G (1 µg/ml; Santa Cruz Biotechnology). After washing with PBS twice, cells were incubated with appropriate secondary antibodies, including Alexa Fluor® 555 goat anti-mouse IgG (2 µg/ml; Invitrogen), Alexa Fluor® 555 rabbit anti-goat IgG (2 µg/ml; Invitrogen), and Alexa Fluor® 488 goat anti-rabbit IgG (2 µg/ml; Invitrogen), in PBS at RT for 1 h. After extensive washing with PBS, the specimens were mounted immediately, and observed using an inverted fluorescence microscope (Nikon Eclipse TE2000-U, Japan) equipped with a CCD camera.

### Statistical analysis

All data were presented as mean ± standard error and analyzed using GraphPad Prism 4.0 software (GraphPad Software). Results were further analyzed using one-way analysis of variance (ANOVA), and post-test was processed using Tukey’s multiple comparison test. *p* < 0.05 was considered statistically significant.

## Results

### Human DDX3 is required for the production of HIV-1 virus

**Figure 1:**
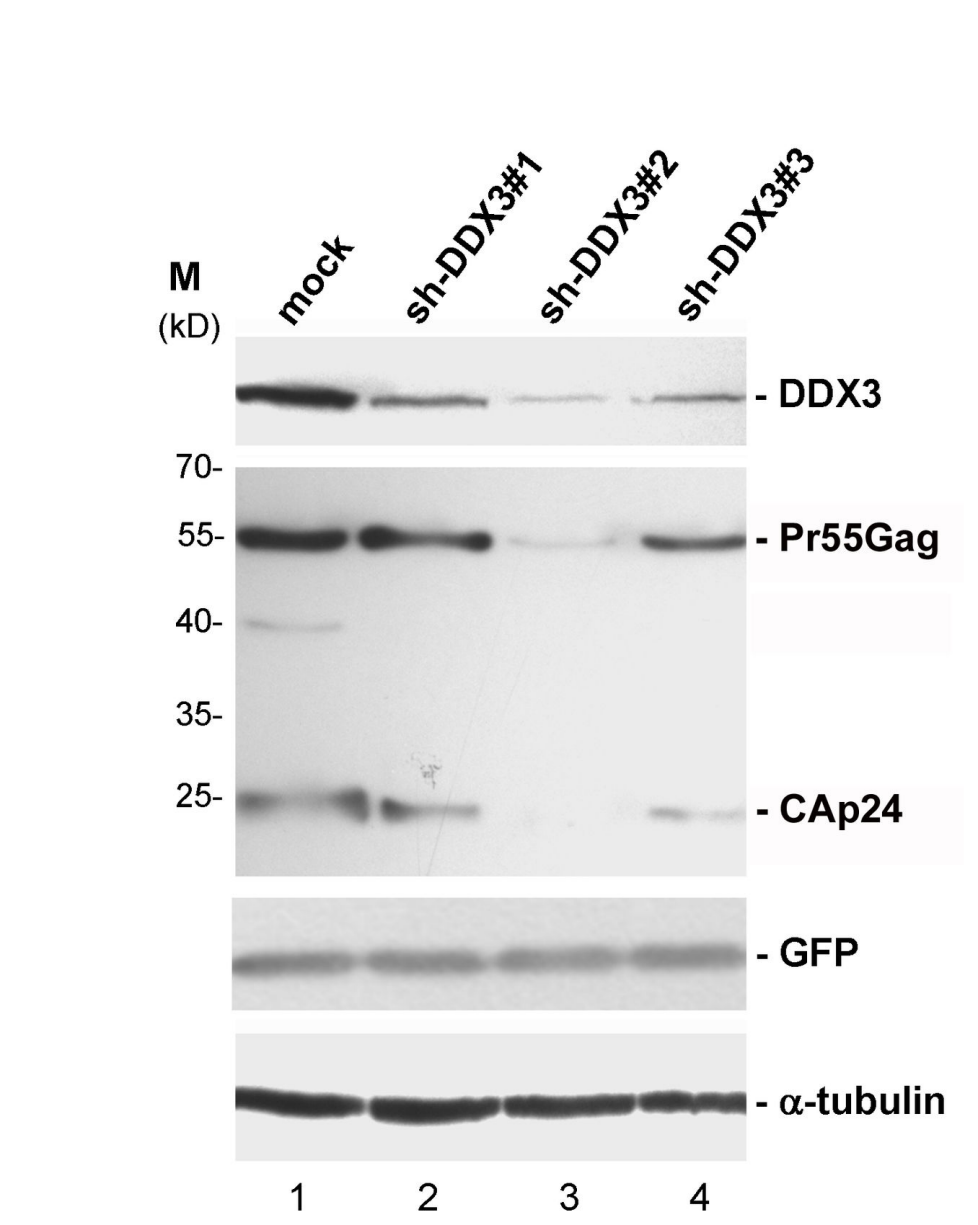
Knockdown of DDX3 by short hairpin RNAs inhibits HIV-1 production. HeLa cells were mock-transfected with empty pSilencer 1.0-U6 vector (lane 1) or transfected with the pSilencer 1.0-U6 vector expressing a shRNA (lanes 2-5), respectively. After 36 hours, cells were re-transfected with HIV-1 proviral DNA pHXB2gpt plasmid, pEGFP-N1 and the same shRNA-expressing vectors. Transfected cells were cultured for another 48 hours, and then harvested for analysis. Immunoblotting was performed using antibodies against DDX3, eIF4A1, HIV-1 p24, GFP and α-tubulin. Viral production was assessed by detecting the expression levels of the HIV-1 Pr55Gag precursor and the mature CAp24 antigen. GFP and α-tubulin served as internal controls.

To investigate the effect of DDX3 on HIV-1 replication, we employed shRNA to knock down DDX3 in HeLa cells, and transfected the HIV-1 proviral DNA plasmid (pHXB2gpt) into these DDX3-depleted HeLa cells. The efficiency of HIV-1 production was detected by the expression of intracellular HIV-1 Pr55Gag precursor and its proteolytic processing byproducts, in particular the mature CAp24 antigen. Immunoblotting showed that the level of DDX3 protein was significantly reduced by three different DDX3-targeting shRNAs (Figure 1, lanes 2-4) of which sh-DDX3#2 was the most effective inhibiting the expression of endogenous DDX3 by ~90% (Figure 1, lane 3). The results also showed that HIV-1 CAp24 production was dramatically reduced by more than 90% in DDX3-depleted HeLa cells (Figure 1, lane 3). This correlation between DDX3 and HIV-1 CAp24 expression indicated that DDX3 is required for HIV-1 production.

### DDX3 facilitates the translation of HIV-1 mRNAs via their 5’ UTRs

**Figure 2:**
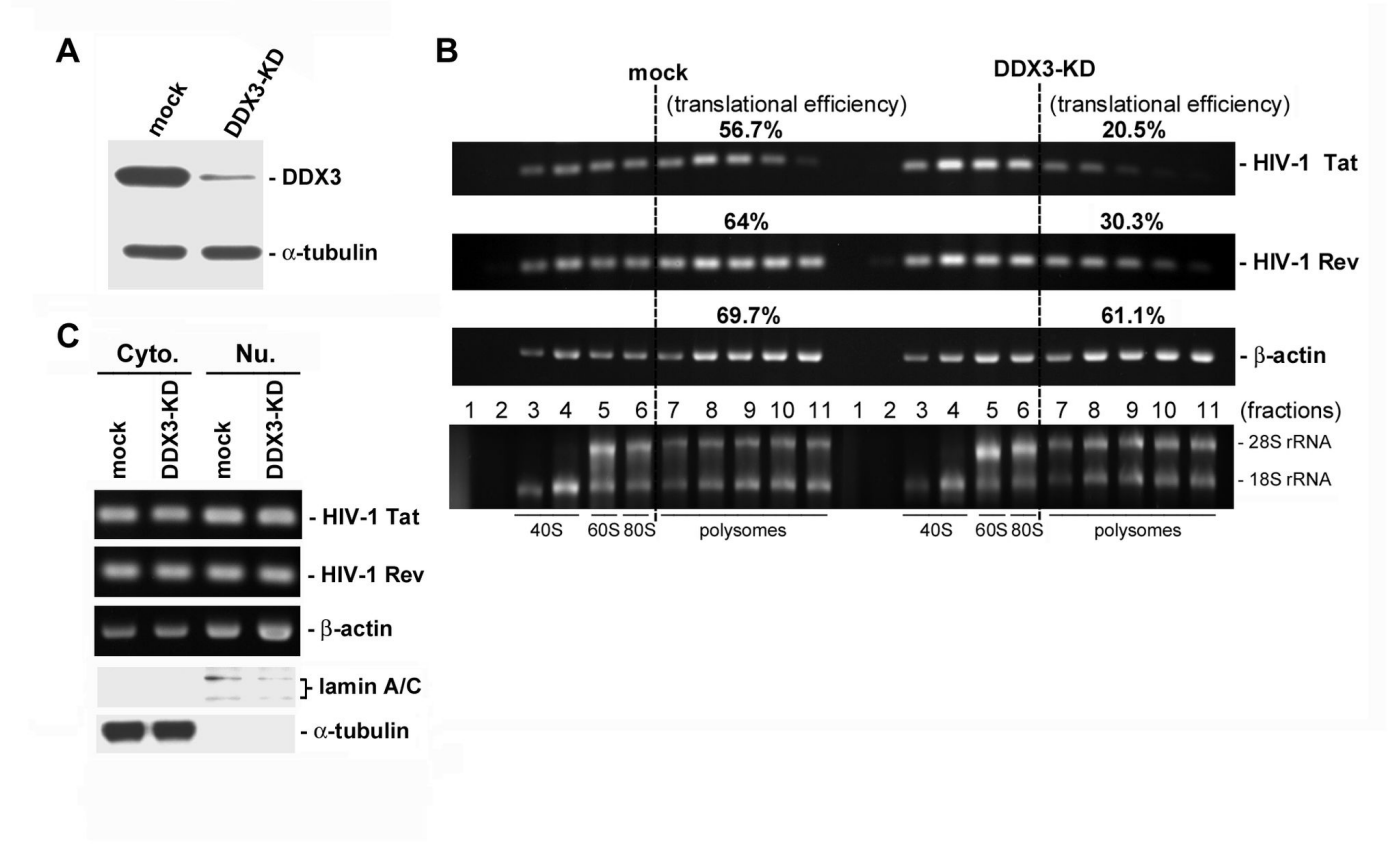
Translation of HIV-1 mRNAs is impaired in DDX3-depleted cells. HeLa cells were transfected with empty pSilencer 1.0-U6 vector (mock) or the pSilencer 1.0-U6 vector expressing sh-DDX3#2 (DDX3-KD). After 72 hours, cells were re-transfected with the proviral DNA pHXB2gpt plasmid and harvested at 12 h post-transfection. **A**. Immunoblotting was performed using anti-DDX3 and anti-α-tubulin antibodies to show the knockdown efficiency of DDX3 in HeLa ***cells*** *. B*. Cytoplasmic extracts prepared from ***mock-transfected*** (mock) or DDX3-depleted (DDX3-KD) HeLa cells were subjected to 15-40% sucrose gradient sedimentation. RNA extracted from gradient fractions was analyzed by conventional RT-PCR using specific primers for HIV-1 *Tat* and *Rev* mRNAs (upper two panels). The housekeeping gene *β-actin* mRNA, whose translation is not significantly affected by DDX3 knockdown, served as a negative control (the 3^rd^ panel). The translational efficiency of each mRNA was calculated as the ratio of polysome-associated mRNAs (fractions 7-11) to total mRNA (all fractions). The 18S and 28S rRNAs were resolved on a 1% formaldehyde/agarose gel and visualized by ethidium bromide staining (lower panel). **C**. RNA extracted from the cytoplasmic (Cyto.) and nuclear (Nu.) fractions of mock-transfected (mock) and DDX3-depleted (DDX3-KD) HeLa cells was analyzed by conventional RT-PCR using specific primers for HIV-1 *Tat*, HIV-1 *Rev*, and *β-actin* mRNAs (upper three panels). The subcellular fractions were also subjected to immunoblotting using anti-lamin A/C and anti-α-tubulin (lower two panels).

To examine whether DDX3 is involved in the translation of HIV-1 mRNAs, we assessed the translational efficiencies of HIV-1 mRNAs in mock-transfected and DDX3-depleted cells using a sucrose gradient sedimentation analysis. Endogenous DDX3 was depleted using sh-DDX3#2 in the following experiments. Immunoblotting showed that the protein level of DDX3 was significantly reduced by ~90% in HeLa cells by sh-DDX3#2 (Figure 2A, DDX3-KD). The polysomal distribution of HIV-1 *Tat* and *Rev* mRNAs was evidently changed in DDX3-depleted cells as compared to the mock cells (Figure 2B). The distribution of an mRNA within the polysomal fractions is reflective of its translational efficiency. A large portion of HIV-1 *Tat* (56.7%) and *Rev* (64%) mRNAs was associated with polysomes in the mock cells, whereas only 20.5% and 30.3% of *Tat* and *Rev* mRNAs remained associated with polysomes in DDX3-depleted cells (Figure 2B). In contrast, the distribution of *β-actin* mRNA in polysomes was not significantly affected by DDX3 knockdown (Figure 2B). Both HIV-1 Tat and Rev are early regulatory proteins, whose mRNAs are fully spliced and exported to the cytoplasm via the TAP-mediated export pathway [12]. Thus, the suppressive effects of DDX3 knockdown on the Rev/RRE/CRM1-mediated export of intron-containing HIV-1 mRNAs could be ruled out. A quantitative assay revealed that the level of HIV-1 *Tat* and *Rev* mRNAs in the cytoplasm was only marginally reduced in DDX3-depleted cells (Figure 2C). Our data therefore support DDX3 being involved in the translation of HIV-1 mRNAs.

**Figure 3:**
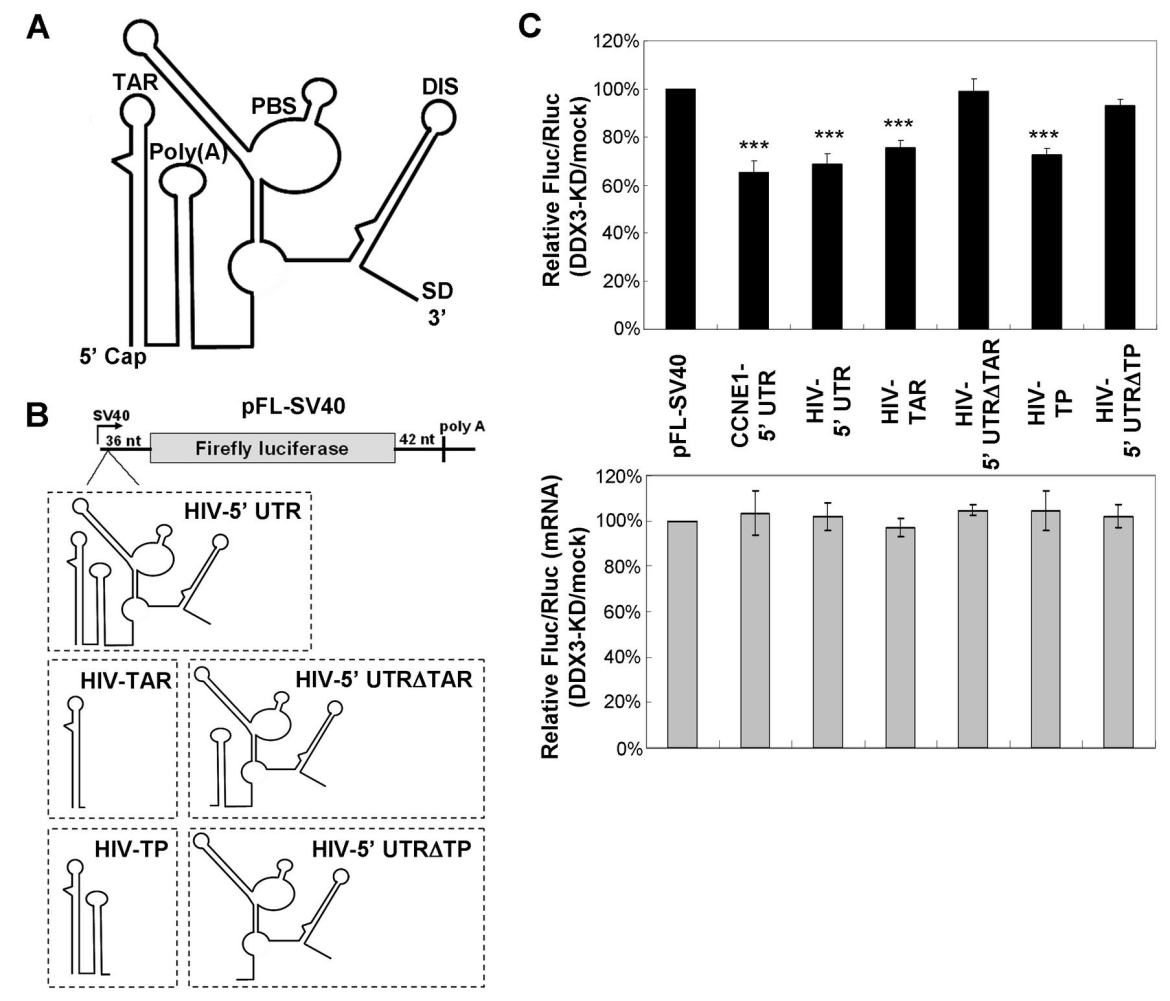
DDX3 facilitates translation of reporter mRNAs containing the 5’ UTR of HIV-1 mRNAs. **A**. Schematic representation of the 5’ UTR of HIV-1 mRNAs. The 5’ UTR of all HIV-1 transcripts shares the same 289 nt noncoding region, which contains numerous cis-acting elements, including the transactivation response (TAR) hairpin, the polyadenylation (poly(A)) hairpin, the primer binding site (PBS), the dimerization initiation site (DIS), and the major splice donor site (SD). RNA secondary structures in the 5’ UTR of HIV-1 mRNA were adapted from Wilkinson et al. [24]. The functional motifs are indicated above each stem-loop domain. **B**. Schematic representation of the firefly luciferase (Fluc) reporters and details on the 5’ UTR of the pFL-SV40 derived reporters. HIV-5’ UTR represents the complete 5’ UTR of HIV-1 mRNAs (nucleotides 1-289). HIV-TAR contains the TAR hairpin (nucleotides 1-57), while HIV-5’ UTRΔTAR encompasses the 5’ UTR devoid of the TAR hairpin (nucleotides 58-289). HIV-TP contains the TAR-poly(A) region (nucleotides 1-104), while HIV-5’ UTRΔTP encompasses the 5’ UTR devoid of the TAR-poly(A) region (nucleotides 105-289). **C**. HeLa cells were co-transfected with a pFL-SV40 derived reporter and the control pRL-SV40 vector encoding the *Renilla* luciferase (Rluc) together with empty pSilencer 1.0-U6 vector (mock) or the pSilencer 1.0-U6 vector expressing sh-DDX3#2 (DDX3-KD) for 48 h. For each transfectant, the Fluc activity was first normalized to that of the Rluc control. Normalized Fluc activity of sh-DDX3#2-transfectants (DDX3-KD) was then compared to that of the mock. The bar graph shows the relative Fluc/Rluc activities in DDX3-KD cells relative to mock cells (upper panel). The pFL-SV40 derived reporter containing the 5’ UTR of *cyclin E1* (CCNE1) served as a positive control. Fluc and Rluc mRNAs were analyzed by quantitative RT-PCR (lower panel). All data are shown as mean (± SEM) from at least three independent experiments (* *p* < 0.05, ** *p* < 0.01, *** *p* < 0.001).

We next investigated whether DDX3 can facilitate the translation of mRNAs with the 5’ UTR of HIV-1 mRNAs, using a dual-luciferase reporter assay. All of the spliced and unspliced HIV-1 transcripts share the same 289 nt 5’ noncoding region (Figure 3A), which contains several structured RNA elements, including the TAR hairpin, the polyadenylation (poly(A)) hairpin, the primer binding site (PBS), and the dimerization initiation site (DIS). The firefly luciferase (Fluc) reporter plasmids containing the complete HIV-1 5’ UTR (289 nt), or portions of this 5’ UTR were constructed (Figure 3B). A *Renilla* luciferase (Rluc) reporter with a non modefied 5’ UTR was co-transfected with the Fluc reporters as an internal control. Changes in translation of reporter mRNAs were assessed by the relative Fluc/Rluc activity in DDX3-depleted cells as compared to the mock cells. As expected, depletion of DDX3 reduced the expression of the HIV-1 5’ UTR reporter by ~30% (Figure 3C, upper panel) without changing the level of the reporter mRNAs (Figure 3C, lower panel), suggesting that DDX3 participates in translational control of HIV-1 mRNAs, at least in part, via the highly structured 5’ UTR. A similar result was obtained from the reporter containing *cyclin E1* 5’ UTR (Figure 3C, CCNE1-5’ UTR), whose translation is closely regulated by DDX3 [8]. To determine the impact of the structural elements within the HIV-1 5’ UTR on translation, the Fluc reporters containing the TAR hairpin (HIV-TAR) and the part of the 5’ UTR without TAR (HIV-5’ UTRΔTAR) were constructed (Figure 3B). The TAR-poly(A) region (HIV-TP) and the part of the 5’ UTR without TAR-poly(A) region (HIV-5’ UTRΔTP), which encompasses an internal ribosome entry site (IRES) [37,38], were also introduced into the 5’ end of the Fluc reporter mRNA (Figure 3B). Luciferase reporter assays showed that depletion of DDX3 reduced the expression of the HIV-TAR and the HIV-TP reporters by ~24% and ~27%, respectively (Figure 3C, upper panel). In contrast, translation of reporters containing the 5’ UTR devoid of the TAR hairpin and the TAR-poly(A) region was not significantly affected by DDX3 knockdown (Figure 3C, upper panel). Our results suggested that the TAR hairpin located at the 5’-end of all HIV-1 transcripts plays a crucial role in DDX3-mediated translational control of HIV-1 mRNAs. In addition, the poly(A) hairpin may also provide a partial contribution in this regard.

### The RNA helicase activity of DDX3 is required for its function in HIV-1 mRNA translation

**Figure 4:**
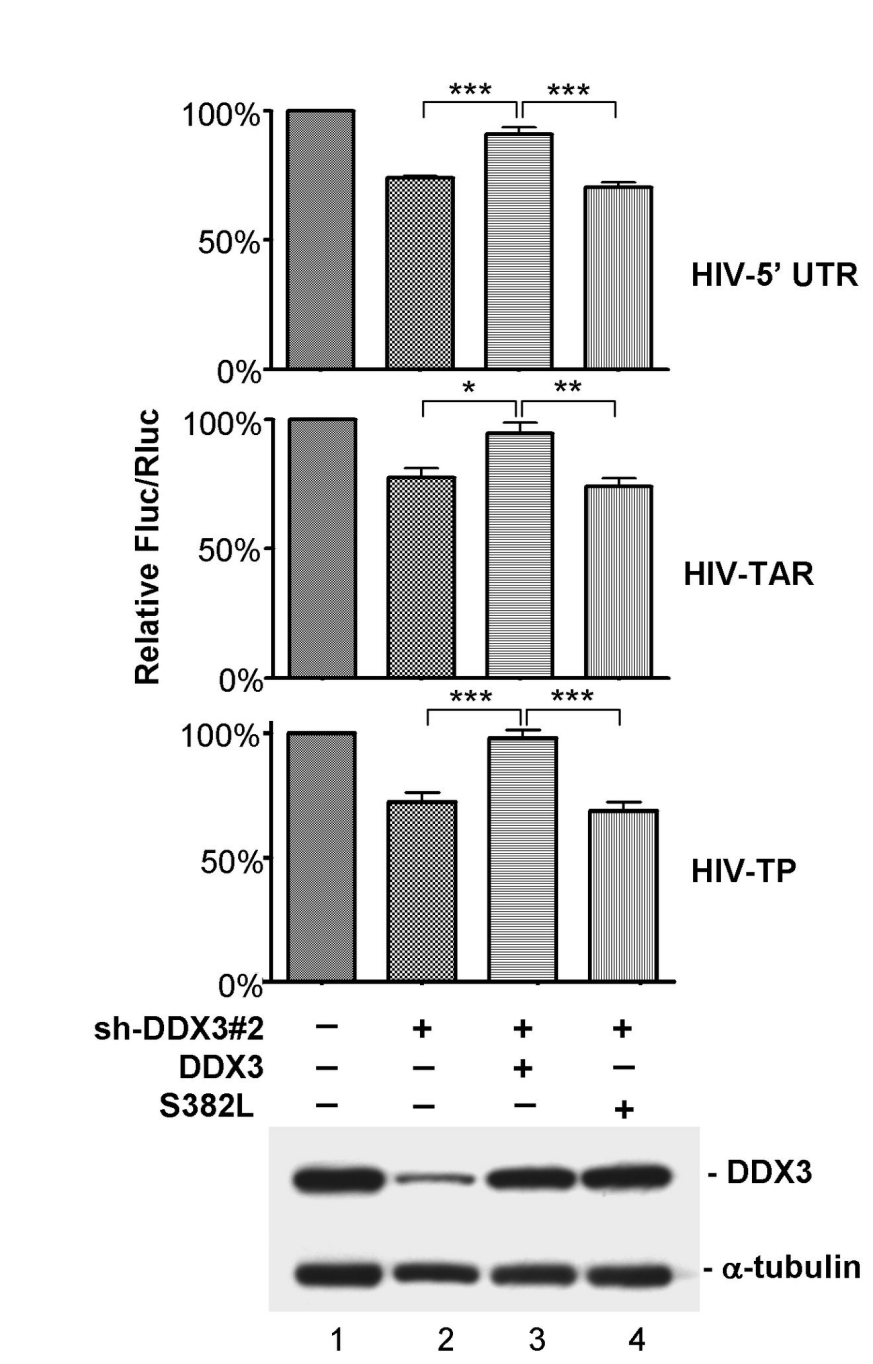
The RNA helicase activity of DDX3 is required for translation of reporter mRNAs containing the HIV-1 5’ UTR. HeLa cells were co-transfected with a pFL-SV40 derived reporter (HIV-5’ UTR, HIV-TAR or HIV-TP) and the control pRL-SV40 vector together with empty pSilencer 1.0-U6 vector (lane 1) or the pSilencer 1.0-U6 vector expressing sh-DDX3#2 (lanes 2-4) and a vector expressing shRNA-resistant wild-type DDX3 (lane 3) or S382L mutant (lane 4) for 48 h. For each transfectant, the Fluc activity was normalized to that of the Rluc control. The bar graph shows the relative Fluc/Rluc activities of each transfection relative to that of the mock transfection (lane 1). All data are shown as mean (± SEM) from at least three independent experiments (* *p* < 0.05, ** *p* < 0.01, *** *p* < 0.001). Immunoblotting was performed using anti-DDX3 and anti-α-tubulin, and representative data is shown (lower panel).

The above-mentioned results suggested that DDX3 may facilitate HIV-1 mRNA translation by resolving secondary structures in their 5’ UTRs. To test this possibility, we performed rescue assays with shRNA-resistant DDX3 constructs to determine whether the RNA helicase activity of DDX3 is required for translational control of HIV-1 mRNAs. Each of the Fluc reporters, including the HIV-1 5’ UTR (HIV-5’ UTR), the TAR hairpin (HIV-TAR), and the TAR-poly(A) region (HIV-TP) was co-transfected with the control pRL-SV40 vector together with sh-DDX3#2 and shRNA-resistant DDX3 constructs (Figure 4). Luciferase reporter assays showed that the translational defect caused by DDX3 knockdown (Figure 4, lane 2) could be rescued by wild-type DDX3 (Figure 4, lane 3), indicating the specificity of sh-DDX3#2. In contrast, a DDX3 mutant (S382L) that has been shown to lose its helicase activity [16] failed to restore the suppressive effects of DDX3 knockdown on the translation of these Fluc reporters (Figure 4, lane 4). We therefore concluded that the RNA helicase activity of DDX3 is required for efficient translation of HIV-1 mRNAs.

#### DDX3 interacts with HIV-1 Tat *in* *vitro* and *in* *vivo*

**Figure 5:**
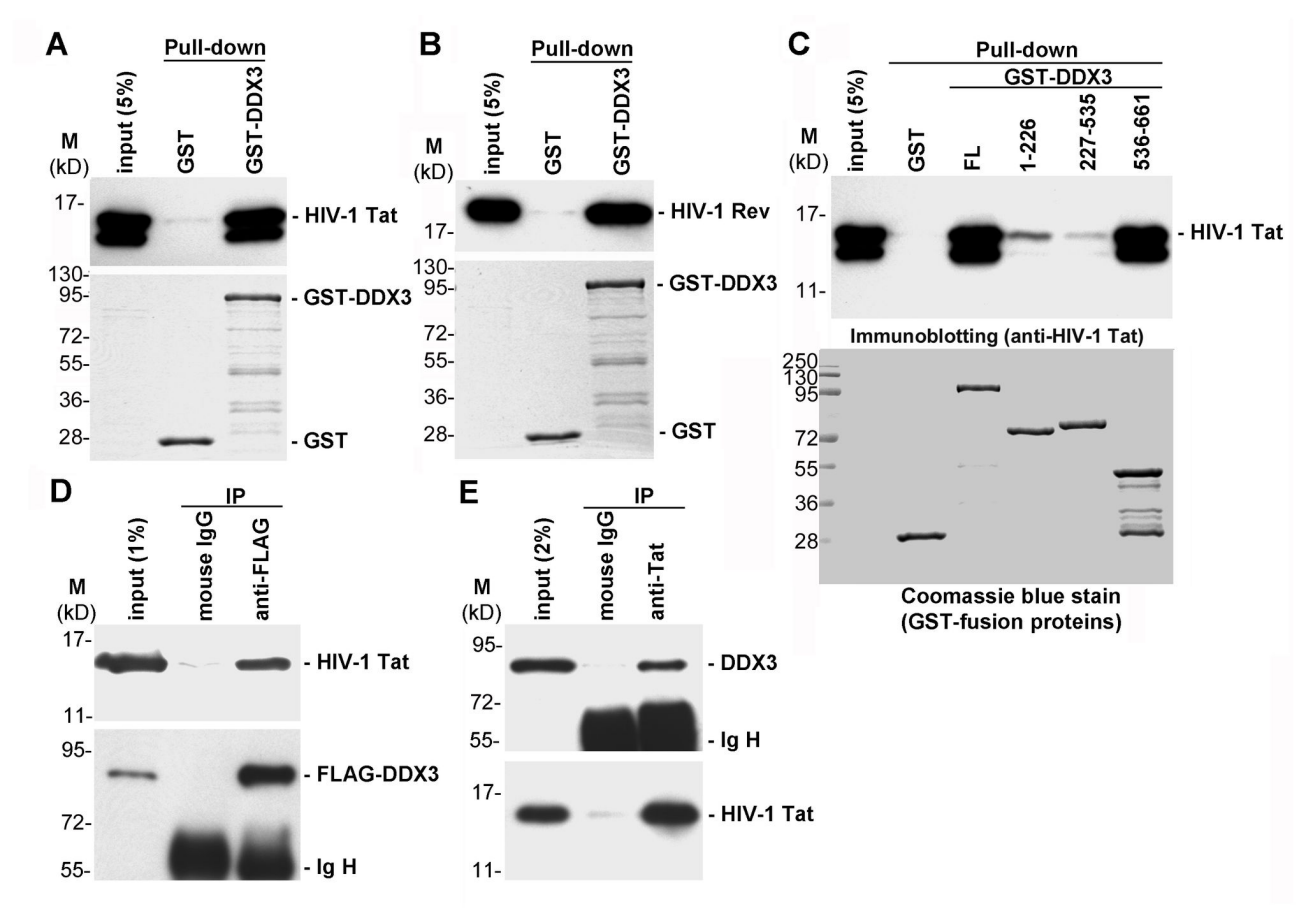
DDX3 interacts with HIV-1 Tat *in vitro* and *in vivo*. **A**. His-tagged recombinant HIV-1 Tat protein was incubated with GST or GST-DDX3. ***After*** GST pull-down, bound proteins were analyzed by immunoblotting with anti-HIV-1 Tat antibody (upper panel). The GST-fusion proteins were resolved by SDS-PAGE and visualized by Coomassie blue staining (lower panel). **B**. The experiment was essentially similar to panel A, except that His-tagged recombinant HIV-1 Rev protein was used in the GST pull-down assay. Bound proteins were analyzed by immunoblotting with anti-HIV-1 Rev antibody (upper panel). **C**. The experiment was essentially similar to panel A. The assay used recombinant GST, GST-DDX3 (full-length; FL) or GST-DDX3 fragments (amino acids 1-226, 227-535 and 536-661) as bait to pull down His-tagged recombinant HIV-1 Tat protein. Bound proteins were analyzed by immunoblotting with anti-HIV-1 Tat antibody (upper panel). The GST-fusion proteins were resolved by SDS-PAGE and visualized by Coomassie blue staining (lower panel). **D**. FLAG-tagged DDX3 and HIV-1 Tat proteins were transiently co-expressed in HEK293 cells for 48 h. Immunoprecipitation was performed using anti-FLAG M2 agarose. Precipitated proteins were subjected to immunoblotting with anti-HIV-1 Tat antibody (upper panel) or anti-DDX3 antibody (lower panel). Ig H represents the immunoglobulin heavy chain. **E**. HIV-1 Tat protein was transiently expressed in HEK293 cells for 48 h. Immunoprecipitation was performed using anti-HIV-1 Tat antibody bound to protein A sepharose beads. Precipitated proteins were subjected to immunoblotting with anti-DDX3 antibody (upper panel) or anti-HIV-1 Tat antibody (lower panel).

Viruses usually take advantage of cellular machineries to facilitate their gene expression and replication. We here provide evidence that DDX3 plays a role in translational control of HIV-1 mRNAs. However, the interplay between DDX3 and viral proteins involved in HIV-1 mRNA translation remains to be explored. Notably, transient expression of HIV-1 Tat induced the expression of DDX3 mRNA in HeLa cells [16]. It has been reported that HIV-1 Tat enhances the translation of TAR-containing mRNA by counteracting the inhibitory effect of the TAR hairpin [39]. We therefore hypothesized that HIV-1 Tat may participate in DDX3-mediated translational control of HIV-1 mRNAs. To test this hypothesis, we first performed a glutathione-S-transferase (GST) pull-down assay to examine whether DDX3 interacts with HIV-1 Tat. His-tagged recombinant HIV-1 Tat protein was incubated with either GST or GST-DDX3. In line with our hypothesis, HIV-1 Tat bound to GST-DDX3 but not GST (Figure 5A). This indicates that DDX3 can directly interact with HIV-1 Tat *in vitro*. Consistent with a previous report [16], recombinant HIV-1 Rev protein was also pulled down by GST-DDX3 but not GST (Figure 5B). We next used GST pull-down assays to identify the interaction domain in the DDX3 for HIV-1 Tat binding. The recombinant HIV-1 Tat protein was incubated with full-length DDX3 (FL) or a series of truncated DDX3 fragments (Figure 5C), including the N-terminal region (residues 1-226), the conserved central DEAD-box core region (residues 227-535), and the C-terminal region (residues 536-661). Our data showed that HIV-1 Tat was pulled down by full-length DDX3 and the C-terminal region of DDX3 (Figure 5C, upper panel). Therefore, the C-terminal region of DDX3, which contains an arginine/serine (RS) dipeptide-rich domain, is responsible for HIV-1 Tat binding. We also performed immunoprecipitation to confirm the interaction between DDX3 and HIV-1 Tat *in vivo*. FLAG-tagged DDX3 and HIV-1 Tat were transiently co-expressed in HEK293 cells. The association between DDX3 and HIV-1 Tat was demonstrated by immunoprecipitation using anti-FLAG antibody, followed by immunoblotting with anti-HIV-1 Tat and anti-DDX3 antibodies (Figure 5D). This association was further confirmed by a reciprocal immunoprecipitation experiment in which endogenous DDX3 was found to co-precipitate ectopically expressed HIV-1 Tat in HEK293 cells (Figure 5E). All immunoprecipitations were performed in the presence of RNase A, indicating that DDX3 directly interact with HIV-1 Tat *in vivo*.

#### HIV-1 Tat is co-localized with DDX3 in cytoplasmic stress granules (SGs)

**Figure 6:**
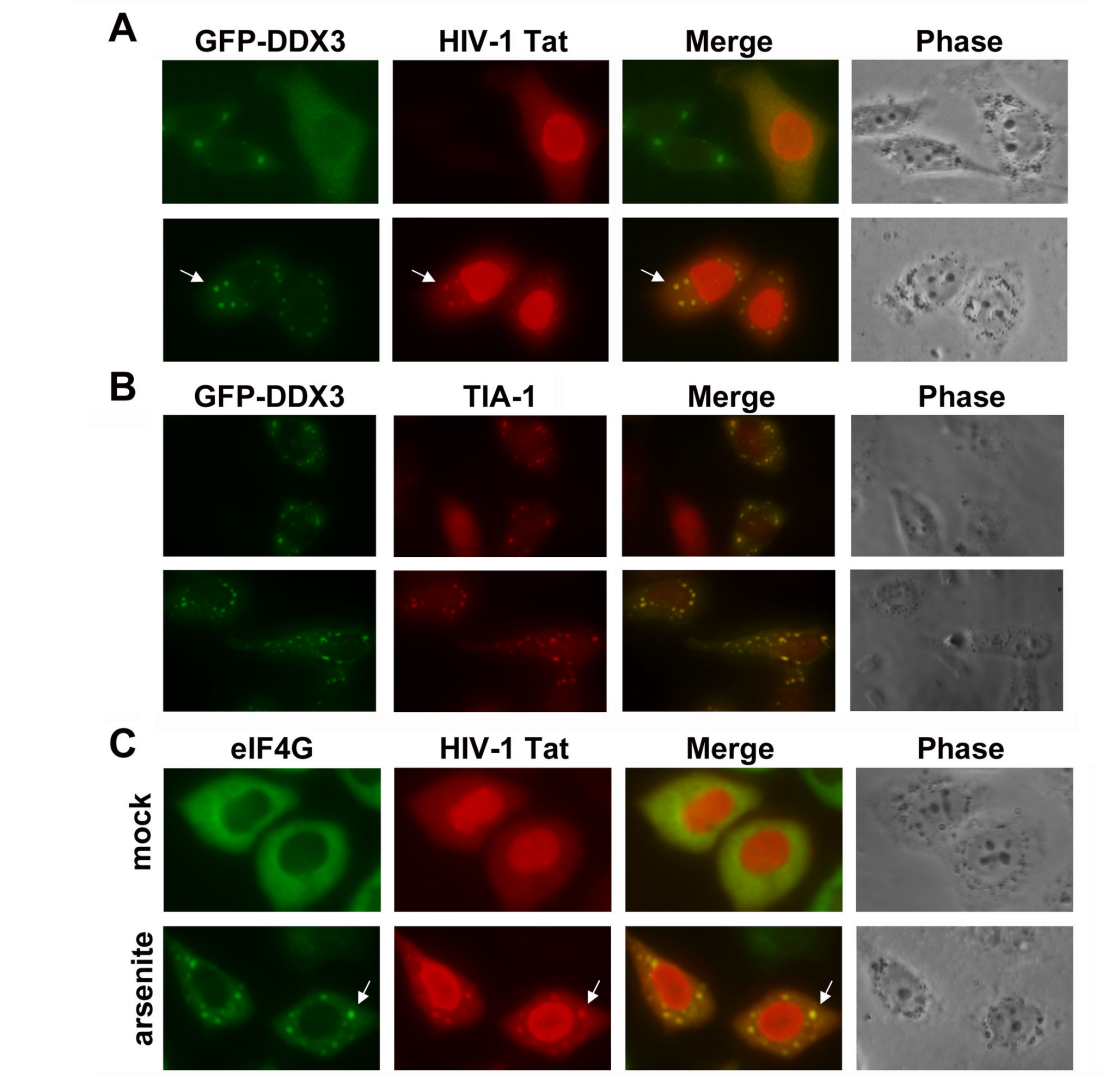
HIV-1 Tat is co-localized with DDX3 in cytoplasmic stress granules under stress conditions. **A**. GFP-DDX3 and HIV-1 Tat were transiently co-expressed in HeLa cells for 24 h. Immunofluorescent staining was carried out using mouse anti-HIV-1 Tat antibody. Subcellular localization of GFP-DDX3 (green) and HIV-1 Tat (red) was observed under a fluorescence microscope. The arrow indicates the co-localization of DDX3 and HIV-1 Tat in cytoplasmic foci. **B**. GFP-DDX3 was transiently expressed in HeLa cells for 24 h. Immunofluorescent staining was carried out using goat anti-TIA-1 antibody. Co-localization of GFP-DDX3 (green) and TIA-1 (red) in cytoplasmic SGs was observed under a fluorescence microscope. **C**. HeLa cells were transfected with a vector expressing HIV-1 Tat protein. At 24 h post-transfection, cells were mock-treated (mock) or treated with 0.5 mM sodium arsenite (arsenite) for 1 h. Immunofluorescent staining was carried out using rabbit anti-eIF4G and mouse anti-HIV-1 Tat antibodies. Subcellular localization of eIF4G (green) and HIV-1 Tat (red) was observed under a fluorescence microscope. The arrow indicates the co-localization of eIF4G and HIV-1 Tat in cytoplasmic SGs.

DDX3 is a nucleo-cytoplasmic shuttling protein and plays multiple functions in the nucleus and cytoplasm. The majority of DDX3 is diffusely distributed throughout the cytoplasm at steady state [6,7]. Under cell stress conditions, DDX3 is largely recruited to cytoplasmic SGs [7,9], suggesting a role of DDX3 in translational control. To further characterize the interaction between DDX3 and HIV-1 Tat, we examined whether HIV-1 Tat can be recruited to cytoplasmic SGs by DDX3. Immunofluorescent staining showed that transient expression of GFP-DDX3 was evenly distributed in the cytoplasm or largely concentrated in cytoplasmic foci (Figure 6A, upper panels), whereas HIV-1 Tat was mainly distributed in the nucleus of transfected HeLa cells. However, HIV-1 Tat was re-localized to cytoplasmic foci when GFP-DDX3 was co-expressed (Figure 6A, lower panels). These cytoplasmic foci were identified as SGs by the accumulation of the SG marker protein TIA-1 (Figure 6B). The subcellular co-localization of HIV-1 Tat and DDX3 further suggests their interaction in the cytoplasm. We also examined whether HIV-1 Tat can be recruited to cytoplasmic SGs under stress conditions. Our results showed that HIV-1 Tat is partially co-localized with the SG marker protein eIF4G in cytoplasmic SGs after sodium arsenite treatment (Figure 6C, lower panels), which induces oxidative stress. SGs are mainly composed of stalled translation pre-initiation complexes, including mRNAs, 40S ribosomal subunits and translation initiation factors. The recruitment of HIV-1 Tat to cytoplasmic SGs suggested a potential role of HIV-1 Tat in translation.

**Figure 7:**
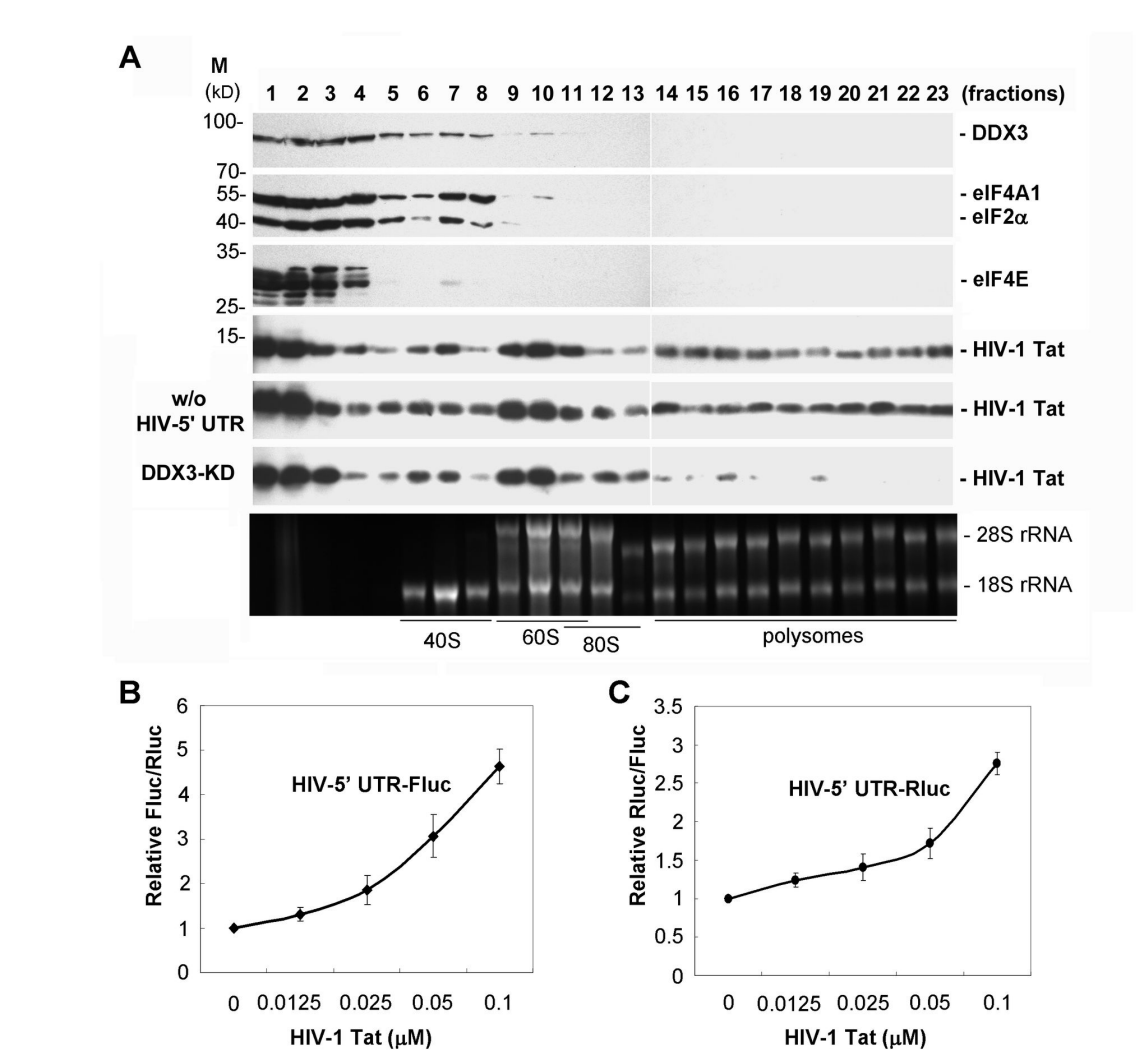
HIV-1 Tat is associated with translating mRNAs and facilitates translation of reporter mRNAs containing the HIV-1 5’ UTR. **A**. HIV-1 Tat protein was transiently co-expressed with the Fluc reporter mRNA containing the 5’ UTR of HIV-1 mRNAs (HIV-5’ UTR) in HEK293 cells for 24 h. Cytoplasmic extracts ***weresubjectedto*** 15-40% sucrose gradient sedimentation. Proteins and RNAs were recovered from 23 fractions for analysis. Immunoblotting analysis of gradient fractions was performed using antibodies against DDX3, eIF4A1, eIF2α, eIF4E and HIV-1 Tat. The association of HIV-1 Tat with translation initiation complexes and polysomes was also detected in the absence of the HIV-1 5’ UTR reporter (w/o HIV-5’ UTR) and in DDX3-depleted (DDX3-KD) HEK293 cells. The 18S and 28S rRNAs were resolved on a 1% formaldehyde/agarose gel and visualized by ethidium bromide staining (lower panel). **B**. The *in vitro* translation assay was performed using *in vitro*-transcribed HIV-1 5’ UTR-containing Fluc mRNA and control Rluc mRNA as templates in HeLa cell lysate supplemented with different amounts of recombinant HIV-1 Tat. The graph shows the relative Fluc/Rluc activities of each reaction relative to that of the corresponding reaction without addition of HIV-1 Tat. All data are shown as mean (± SEM) from at least three independent experiments. **C**. The experiment was essentially similar to panel B, except that Rluc mRNA harbored the HIV-1 5’ UTR while the non-modefied Fluc mRNA served as a control. The graph shows the relative Rluc/Fluc activities of each reaction relative to that of the corresponding reaction without addition of HIV-1 Tat. All data are shown as mean (± SEM) from at least three independent experiments.

Figure 7A, fractions 7-8), but they were undetectable in polysomes (Figure 7A, fractions 14-23). Nevertheless, the HIV-1 Tat protein was associated not only with translation initiation complexes (Figure 7A, fractions 6-13) but also with polysomes (Figure 7A, fractions 14-23), suggesting that HIV-1 Tat remains associated with translating mRNAs in the cytoplasm. We further detected the polysomal association of HIV-1 Tat in the absence of either the HIV-1 5’ UTR reporter or DDX3. Without the HIV-1 5’ UTR reporter (Figure 7A, w/o HIV-5’ UTR), HIV-1 Tat remained associated with polysomes. Importantly, DDX3 knockdown in HEK293 cells resulted in a decrease in the association of HIV-1 Tat with polysomes (Figure 7A, DDX3-KD). This supports the importance of DDX3 in the translational function of HIV-1 Tat. We also examined whether HIV-1 Tat could facilitate translation of reporter mRNAs containing the HIV-1 5’ UTR using an *in vitro* translation assay. Addition of recombinant HIV-1 Tat in HeLa cell lysate showed that translation of the HIV-1 5’ UTR-containing Fluc mRNA but not the control Rluc mRNA was activated in a dose-dependent manner (Figure 7B). A similar result was obtained from a reciprocal translation experiment in which Rluc mRNA harbored the HIV-1 5’ UTR while the Fluc mRNA with a short unstructured 5’ UTR served as a control (Figure 7C). The results indicated that translation of HIV-1 mRNAs is facilitated by the Tat protein. Therefore, HIV-1 Tat may function as a transcriptional activator in the nucleus and subsequently accompany viral mRNAs to the cytoplasm for translation.

## Discussion

Previous reports have indicated that DDX3 is required for the replication of HIV-1 [16,18] and hepatitis C virus (HCV) [40], but the underlying molecular mechanisms remain poorly understood. Herein, we showed that depletion of DDX3 by shRNA inhibited the expression of HIV-1 CAp24 antigen (Figure 1), an indicator of HIV-1 production. DDX3 was thought to facilitate the export of unspliced and partially spliced HIV-1 mRNAs, but not fully spliced mRNAs such as that of *Tat* and *Rev* transcripts. Most importantly, knockdown of DDX3 attenuated the translational efficiencies of the HIV-1 *Tat* and Rev but did not significantly affect their mRNA levels in the cytoplasm (Figure 2). Because HIV-1 Rev is required for the export of intron-containing viral mRNAs to the cytoplasm [12], our data indicate that the impairment of HIV-1 mRNA export observed in DDX3-depleted cells [16,18] is due, at least in part, to the down-regulation of HIV-1 Rev expression. Taken together, our results provide evidence to show that DDX3 facilitates not only the nuclear export but also the protein synthesis of HIV-1 mRNAs, and this has a significant impact on HIV-1 replication in infected host cells.

Likewise, it has been reported that yeast Ded1 is required for the replication of brome mosaic virus (BMV) [30], a model positive-strand RNA virus whose replication has been well-characterized. Yeast Ded1 was shown to function in the 5’ noncoding region of BMV *RNA2*, which encodes viral polymerase-like protein 2a, through a translational mechanism [30]. In addition to DDX3, other DEAD-box proteins also exhibit different expression patterns during HIV-1 replication [41]. For example, DDX1 was identified as a cellular cofactor for the Rev/RRE/CRM1-mediated export of HIV-1 mRNAs [42]. RNA helicase A was also shown to modulate translation of HIV-1 mRNAs and infectivity of progeny virions [43]. Therefore, HIV-1 replication may be intricately regulated by DDX3 together with other RNA helicases.

DDX3 and its homologs are known to facilitate translation of selected mRNAs [7,8,11,30]. We previously demonstrated that DDX3 is required for translation of mRNAs containing a long or structured 5’ UTR [7]. Secondary structures located in the 5’ UTR of eukaryotic mRNAs may inhibit translation initiation by interfering with ribosome scanning. Hairpin thermal stability and cap-to-hairpin distances are critical for the control of translation [44,45]. In particular, translation initiation is highly susceptible to hairpin structures near the 5’ cap [22]. The TAR hairpin located at the cap-proximal region of HIV-1 transcripts indeed exhibits a strong inhibitory effect on translation [25]. Biochemical studies indicated that yeast Ded1 is more potent than eIF4A in regard to the RNA unwinding activity [27] and the processivity of 40S ribosome scanning [31]. Our previous studies also demonstrated that DDX3 promotes translation of selected mRNAs with complex 5’ UTRs via its RNA helicase activity [7,8]. We here show that DDX3 facilitates translation of mRNAs containing the highly structured HIV-1 5’ UTR, especially with the TAR hairpin (Figure 3). Consistently, a recent study showed that DDX3 is required for translation of the HIV-1 genomic RNA [46]. The translational defect caused by DDX3 knockdown could be rescued by wild-type DDX3 but not the RNA helicase mutant (S382L) (Figure 4), suggesting that the RNA helicase activity of DDX3 is required for its function in HIV-1 mRNA translation. Our results support the view that DDX3 functions in ribosome scanning, while eIF4A is thought to be required for binding of 43S pre-initiation complexes to the 5’ cap of mRNAs during translation initiation. However, recent reports also suggest that DDX3 functions in the 43S ribosome binding prior to scanning [46], or the formation of 80S ribosome [47]. The consensus from these studies indicate the essential role of DDX3 in translation initiation. Nevertheless, the precise point of DDX3 action is controversial and remains to be clarified.

HIV-1 and related retroviruses have been shown to initiate translation by both cap-dependent and IRES-mediated mechanisms [23]. Notably, the reporter mRNA with the TAR hairpin of the HIV-1 5’ UTR is more susceptible to DDX3 knockdown than that with the 5’ UTRs devoid of the TAR hairpin (Figure 3C), the latter encompasses an IRES element [37,38]. It is possible that DDX3 facilitates cap-dependent ribosome scanning but not HIV-1 IRES-mediated translation of HIV-1 mRNAs. Although DDX3 has been reported to enhance HIV-1 IRES-mediated translation [48], recent studies indicate that cap-dependent ribosome scanning is the main mechanism for HIV-1 mRNA translation [49,50]. The activity of the HIV-1 IRES is stimulated perhaps only under cell stress conditions [51] or during the G2/M phase of the cell cycle [37]. However, further investigations are needed to understand the requirement of DDX3 for IRES-mediated translation initiation.

Most DEAD-box RNA helicases show no specificity for RNA substrate *in vitro* [52]. Their substrate specificities can be provided by selective interactions with RNA binding proteins. We have recently reported that DDX3 has no RNA binding preference [8], but it facilitates translation initiation of selected mRNAs. Here, we showed that DDX3 directly interacts with HIV-1 Tat *in vitro* and *in vivo* (Figure 5). The C-terminal domain of DDX3, which contains an RS-domain found in a subset of splicing factors [53], is responsible for HIV-1 Tat binding (Figure 5C). We also showed the subcellular co-localization of DDX3 and HIV-1 Tat in cytoplasmic SGs (Figure 6A). Endogenous DDX3 is largely recruited to cytoplasmic SGs under stress conditions, such as oxidative stress or heat shock [7]. Overexpression of DDX3 also induced the formation of SGs in HeLa cells [7], whereas knockdown of DDX3 was shown to interfere with SG assembly [9]. These findings together suggest that DDX3 is a key component of cytoplasmic SGs. Although it has been reported that HIV-1 production prevents the SG assembly induced by arsenite but not puromycin [54], this suggests that HIV-1 Tat might be recruited to some but not all types of SGs during viral replication. Interestingly, we found that HIV-1 Tat is partially recruited to cytoplasmic SGs upon DDX3 overexpression (Figure 6A) or under stress conditions (Figure 6C). Co-localization of DDX3 and HIV-1 Tat in cytoplasmic SGs supports their interaction in the cytoplasm and a potential role of HIV-1 Tat in translation. HIV-1 Tat is a well-characterized transcriptional activator in the nucleus. However, its cytoplasmic functions in translation remain elusive. It has been shown that HIV-1 Tat enhances the translation of TAR-containing mRNA by counteracting the inhibitory effect of the TAR hairpin [39], but the underlying mechanisms are unclear. The HIV-1 Tat protein was also shown to stimulate translation of its cognate mRNA in a TAR-dependent manner [55]. More recently, it was reported that the HIV-1 Tat protein and the structured 5’ UTR of HIV-1 mRNAs can modulate the programmed -1 ribosomal frameshift [50], a HIV-1-specific mechanism required for translation of the Gag-Pol polyprotein. The increase in frameshift efficiency caused by the TAR-poly(A) region was also antagonized by the HIV-1 Tat protein [50], suggesting a regulatory role of HIV-1 Tat in viral translation. Using sucrose gradient sedimentation analysis, we showed that HIV-1 Tat was detected in translation initiation complexes and polysomes (Figure 7A). This suggests that HIV-1 Tat remains associated with translating mRNAs in the cytoplasm. The polysomal association of HIV-1 Tat is dependent on DDX3 (Figure 7A), supporting the role of Tat/DDX3 complex in translation. To rule out the effects of HIV-1 Tat on transcriptional activation, we performed *in vitro* translation assays to further demonstrate that HIV-1 Tat facilitates the translation of mRNA containing the HIV-1 5’ UTR (Figure 7B and C). The events of mRNA metabolism from transcription to translation are tightly coordinated [56]. Therefore, it is an attractive thought that HIV-1 Tat might play a role in integrating these processes to ensure efficient progression of viral mRNAs from transcription to translation. Since DDX3 also functions as a transcriptional regulator in the nucleus [1,2], we cannot completely rule out the possibility that the interaction between DDX3 and HIV-1 Tat may play a role in transcription. Perhaps disrupting the interaction between cellular DDX3 and viral Tat or Rev might be detrimental to HIV-1 replication. Further experiments are needed to resolve the details of the interplay mechanisms between DDX3 and these viral proteins.
